# An antibody against HK blocks Alzheimer’s disease peptide β-amyloid−induced bradykinin release in human plasma

**DOI:** 10.1073/pnas.1914831116

**Published:** 2019-10-28

**Authors:** Zu-Lin Chen, Pradeep Singh, Jyen Wong, Katharina Horn, Sidney Strickland, Erin H. Norris

**Affiliations:** ^a^Patricia and John Rosenwald Laboratory of Neurobiology and Genetics, The Rockefeller University, New York, NY 10065

**Keywords:** Alzheimer’s disease, bradykinin, beta-amyloid, high molecular weight kininogen

## Abstract

Bradykinin is a proinflammatory factor that mediates angioedema and inflammation in many diseases. It is a key player in some types of hereditary angioedema and is involved in septic shock, traumatic injury, Alzheimer’s disease (AD), and stroke, among others. Activation of the plasma contact system leads to elevated levels of plasma kallikrein, which cleaves high molecular weight kininogen (HK) to release bradykinin. Drug development for bradykinin-meditated pathologies has focused on designing inhibitors to the enzymes that cleave HK (to prevent bradykinin release) or antagonists of endothelial bradykinin receptors (to prevent downstream bradykinin action). Here we show a strategy to block bradykinin generation by using an HK antibody that binds to HK, preventing its cleavage and subsequent bradykinin release. We show that this antibody blocks dextran sodium sulfate-induced HK cleavage and bradykinin production. Moreover, while the pathogenic AD peptide β-amyloid (Aβ)42 cleaves HK and induces a dramatic increase in bradykinin production, our HK antibody blocked these events from occurring. These results may provide strategies for developing treatments for bradykinin-driven pathologies.

The plasma contact system is initiated by activation of the protease factor XII (FXII) to FXIIa. Once activated, FXIIa can launch both prothrombotic and proinflammatory pathways (1). FXIIa activation of FXI leads to thrombin generation and fibrin formation, while FXIIa activation of plasma prekallikrein leads to the release of bradykinin via cleavage of the intact form of high molecular weight kininogen (HK) and subsequent activation of inflammatory processes (2).

Bradykinin increases vascular permeability, which leads to vasodilation and edema, upon binding to its receptors on endothelial cells (3). Increasing evidence indicates that elevated contact system activation and increased bradykinin generation are implicated in many pathologies in a variety of systems and organs (4–7). The development of drugs to treat bradykinin-mediated pathologies has been focused on inhibiting enzymes that lead to increased HK cleavage or antagonizing bradykinin receptors. Drugs have not been developed directly against HK, from which bradykinin is released (8). We have generated several HK antibodies (9) that bind HK and block its cleavage, preventing bradykinin release induced by dextran sulfate sodium salt (DXS) or Aβ42. These results suggest that targeting HK cleavage can lead to blockage of bradykinin release. Our studies may provide important information for the development of strategies to treat bradykinin-driven pathologies.

## Results and Discussion

### HK Antibodies Bind HK and Block DXS-Induced HK Cleavage and Bradykinin Release in Human Plasma.

**Fig. 1.**
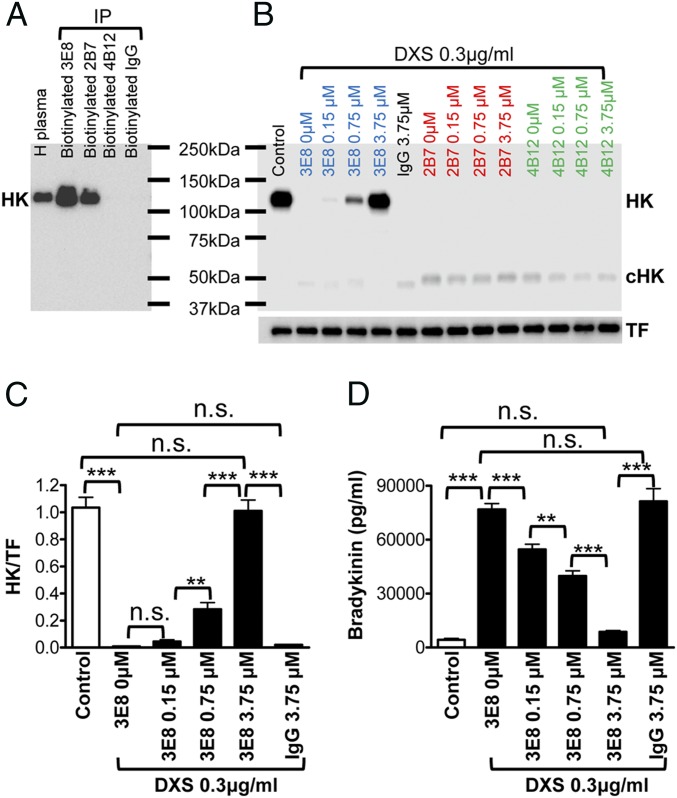
HK antibodies bind HK and block DXS-induced HK cleavage and bradykinin production in human plasma. (*A*) Human plasma was incubated with biotinylated 3E8, 2B7, and 4B12 HK antibodies and control IgG, and streptavidin was added to pull down the antibody−antigen complex. The samples were analyzed by Western blot using a commercial HK antibody. The 3E8 and 2B7 HK antibodies bound HK and immunoprecipitated it from human plasma, while 4B12 HK antibody and control IgG did not pull down HK from human plasma. (*B*) HK Western blot showed 3E8 HK antibody dose-dependently blocked DXS-induced HK cleavage. Without HK antibodies, DXS induced complete cleavage of HK (disappearance of HK band and appearance of cHK band). The 2B7 and 4B12 HK antibodies and control IgG did not show any effects on HK cleavage. (*C*) The protective effects of 3E8 antibody were quantified by measuring the intensity of HK bands after normalization to TF. The 3E8 at a higher concentration (3.75 μM) completely blocked DXS-induced HK cleavage, and 3E8 at lower concentration (0.75 μM) also significantly blocked HK cleavage. Control IgG did not show any effect on HK cleavage. (*D*) The effects of 3E8 antibody on DXS-induced bradykinin production were measured by ELISA. DXS induced a dramatic increase in bradykinin production, but 3E8 at 3.75 μM completely blocked DXS-induced bradykinin production. The 3E8 at lower concentrations (0.15 or 0.75 μM) also significantly blocked bradykinin production. Control IgG did not show any effects on DXS-induced bradykinin production. Data are denoted as mean ± SD. **P ≤ 0.01, and ***P ≤ 0.001. *P* > 0.05 was not significant (n.s.).

We generated monoclonal antibodies that recognize HK (2B7) or cleaved HK (cHK, 4B12), or both (HK/cHK, 3E8) (9). To investigate whether these antibodies bind HK in human plasma and protect HK from cleavage, we performed immunoprecipitation experiments. The 3E8 and 2B7 antibodies pulled down HK from human plasma, while 4B12 and control IgG did not (Fig. 1*A*), indicating 3E8 and 2B7 antibodies bind HK in human plasma. We next determined whether the binding of 3E8 and 2B7 to HK in plasma could prevent HK cleavage. We added DXS, a negatively charged chemical that activates the contact system (10), to human plasma in the absence or presence of 3E8, 2B7, or 4B12 HK antibodies. In the absence of HK antibodies, DXS completely cleaved HK (Fig. 1*B*). While 3E8 at low doses (0.15 μM or 0.75 μM) only partially blocked DXS-induced HK cleavage, a higher dose (3.75 μM) completely blocked cleavage. Antibodies 2B7, 4B12, and control IgG did not influence DXS-induced HK cleavage (Fig. 1*B*). We quantified the effects of 3E8 on DXS-induced HK cleavage (after normalization to transferrin [TF]) and found that 0.15 μM 3E8 did not affect DXS-induced HK cleavage, but 0.75 μM 3E8 significantly blocked DXS-induced HK cleavage and 3.75 μM 3E8 completely blocked HK cleavage, as HK levels were consistent with that of controls (Fig. 1*C*).

We further analyzed the effect of 3E8 on DXS-induced bradykinin release. DXS (0.3 μg/mL) increased bradykinin levels, but 3E8 HK antibody dose-dependently blocked DXS-induced bradykinin production (Fig. 1*D*). At 3.75 μM, 3E8 completely blocked DXS-induced bradykinin release. Control IgG did not show any effect on DXS-induced bradykinin production. These results indicate that 3E8 dose-dependently blocks DXS-induced HK cleavage and bradykinin production.

### HK Antibodies Block Aβ42-Induced HK Cleavage and Bradykinin Release in Human Plasma.

**Fig. 2.**
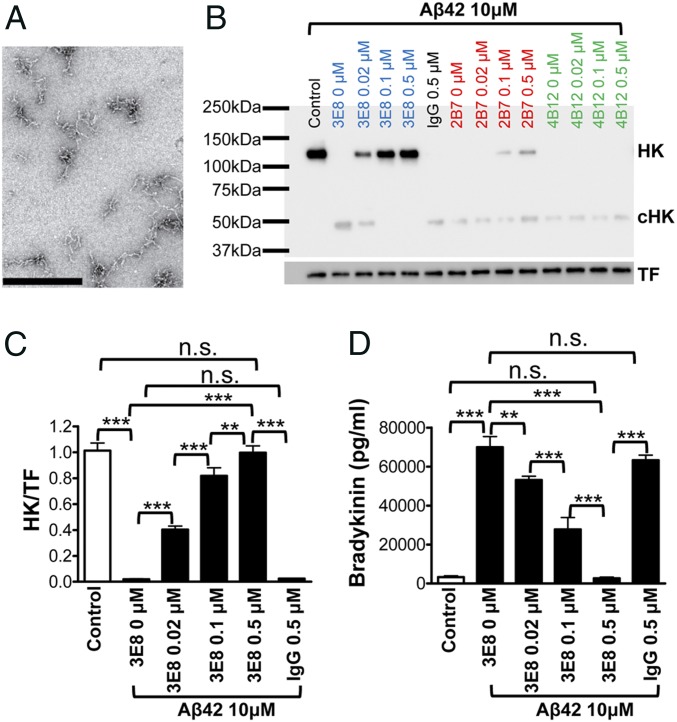
HK antibodies block Aβ42-induced HK cleavage and bradykinin release in human plasma. (*A*) Representative transmission electron microscopy image of Aβ42 used in this study. (Scale bar, 500 nM.) (*B*) HK Western blot shows that, in the absence of HK antibodies, Aβ42 completely cleaved HK. However, the 3E8 HK antibody dose-dependently blocked Aβ42-induced HK cleavage. The 2B7 antibody (at 0.1 and 0.5 μM) also showed a limited protective effect against HK cleavage. The 4B12 HK antibody and control IgG did not show any effects on HK cleavage. (*C*) The protective effects of 3E8 HK antibody were quantified as described in Fig. 1. The 3E8 antibody dose-dependently blocked Aβ42-induced HK cleavage, and, at 0.5 μM, it completely blocked HK cleavage. Control IgG did not show any effect on HK cleavage. (*D*) The effects of 3E8 antibody on Aβ42-induced bradykinin production were measured by bradykinin ELISA. Aβ42 induced a dramatic increase in bradykinin production, but the addition of 3E8 (0.5 μM) completely blocked Aβ42’s effects on bradykinin generation. The 3E8 at 0.02 or 0.1 μM also significantly blocked bradykinin production. Control IgG did not show any effects on Aβ42-induced bradykinin production. Data are denoted as mean ± SD. **P ≤ 0.01, ***P ≤ 0.001. *P* > 0.05 was not significant (n.s.).

Plasma contact system activation is involved in Alzheimer’s disease, and the pathological peptide, Aβ42, can induce plasma contact system activation and HK cleavage (11, 12). We examined whether our HK antibodies could block Aβ42-induced HK cleavage. In the absence of HK antibodies, Aβ42 protofibrils (Fig. 2*A*) induced complete cleavage of HK. Low doses of 3E8 HK antibody (0.02 μM or 0.1 μM) partially blocked Aβ42-induced HK cleavage, but a higher dose (0.5 μM) completely blocked cleavage (Fig. 2*B*). The 2B7 antibody showed a limited effect on Aβ42-induced HK cleavage, and 4B12 and control IgG did not show any effect on Aβ42-induced HK cleavage. We quantified the effect of 3E8 on Aβ42-induced HK cleavage by measuring the intensity of HK bands (Fig. 2*C*). The 3E8 dose-dependently blocked Aβ42-induced HK cleavage, and completely blocked cleavage at 0.5 μM.

We also analyzed whether Aβ42-induced HK cleavage leads to bradykinin production and whether blocking HK cleavage by 3E8 prevents bradykinin release. Aβ42 (10 μM) induced a dramatic release of bradykinin, and 3E8 dose-dependently blocked Aβ42’s effects (Fig. 2*D*). At 0.5 μM, 3E8 completely blocked Aβ42-induced bradykinin release. Control IgG did not show any effect on Aβ42-induced bradykinin release. These results show that 3E8 dose-dependently blocked Aβ42-induced HK cleavage as well as bradykinin production in human plasma.

Our results show that 3E8 and 2B7 HK antibodies can bind HK in human plasma and prevent HK cleavage. The 3E8 antibody is more efficient than 2B7, likely due to the differences in their binding sites on HK or their binding affinities. The 2B7 only showed a limited protective effect on Aβ42-induced HK cleavage and did not show any effect on DXS-induced HK cleavage, possibly because DXS is a stronger activator of the contact system and 2B7 is a weak inhibitor of HK cleavage. Consistent with this idea, a higher concentration of 3E8 was needed to completely block DXS-induced HK cleavage (3.75 μM) than the concentration needed to block Aβ42-induced HK cleavage (0.5 μM). Since the 4B12 antibody only recognizes cHK, it did not have any effect on Aβ42- or DXS-induced HK cleavage.

Plasma contact system activation and increased bradykinin release are involved in pathophysiology of many diseases (4, 13). Our results suggest that antibodies targeting HK and blocking its cleavage may provide a promising strategy to prevent bradykinin release and subsequent edema and inflammation. Further studies regarding this strategy may lead to better therapeutics to treat bradykinin-mediated diseases.

## Materials and Methods

Blood was collected into (ethylenedinitrilo)tetraacetic acid-coated tubes and plasma prepared from healthy human donors (*n* = 3) who provided informed consent. The research study was approved by The Rockefeller University Institutional Review Board. Preparation of Aβ42 (Anaspec) and transmission electron microscopy method were described previously (9, 12). HK antibodies (9) and control IgG (Innovative Research) were biotinylated using EZ-Link Sulfo-NHS-LC-Biotin (Thermo Scientific). Plasma was incubated with biotinylated HK antibodies and control IgG. Dynabeads M-280 Streptavidin (Invitrogen) was used to pull down the antibody−antigen complex (9). Samples were eluted with sodium dodecyl sulfate sample buffer, and Western blots were performed.

To analyze the effects of HK antibodies on DXS-induced plasma HK cleavage, plasma was incubated with HK antibodies or control IgG at varying concentrations at 37 °C for 20 min, and then 0.3 μg/mL DXS was added and incubated for 1 h at 37 °C. To investigate the effects of HK antibodies on Aβ42-induced plasma HK cleavage, plasma was incubated with HK antibodies or control IgG at 0, 0.02, 0.1, or 0.5 μM at 37 °C for 20 min, and then 10 μM Aβ42 was added and incubated for 2 h at 37 °C. HK cleavage was analyzed by Western blot, and bradykinin concentrations were determined by enzyme-linked immunosorbent assay (ELISA).

Western blots were performed as described previously (9, 12). Anti-human HK antibody (Abcam) and anti-TF antibody (Abcam) were used. Blots were imaged via Bio-Rad ChemiDoc. Protein levels were quantified by densitometry with NIH Image J. Plasma bradykinin ELISA (Enzo Life Sciences) was performed according to manufacturer’s instructions.

All experiments were performed in duplicate and repeated at least 3 times. All statistical analyses were performed using GraphPad Prism 4 software. Comparisons among multiple groups were performed using one-way ANOVA followed by Newman−Keuls multiple comparison test.
